# Supplementary material for: Adaptive experimental design produces superior and more efficient estimates of predator functional response
Source: PLoS One. 2023 Jul 20;18(7):e0288445. doi: 10.1371/journal.pone.0288445 (PMC10358903; doi:10.1371/journal.pone.0288445)
Supplement: S1 Appendix — pdf of supplementary document referred to in the main text. (PDF) [file pone.0288445.s001.pdf]

Supplementary Document for: Adaptive experimental design produces superior and more efficient estimates of predator functional response

1 Sequential Experimental Design

In this section we describe the sequential experimental design procedure that we use for functional response experiments, which can also be adapted to conduct sequential design in other applications. The description closely follows that in Moffat et al. (2020), which performed simulation studies and no real experiment.

1.1 Bayesian Formulation

Define K to be the total number of candidate predator-prey functional response models under consideration. In the main paper we have $K = 2$, and the models are given by the Type II or Type III functional response models, each with a beta-binomial response distribution. Let $M \in \{1, \dots, K\}$ be a random variable that indicates which model is responsible for data generation. Let $n_{1:t} = (n_1, n_2, \dots, n_t)$ denote a vector of observed data where n_j is the number of prey consumed in experiment j. Let the corresponding number of initial prey for the t experiments be $N^0_{1:t} = (N^0_1, N^0_2, \dots, N^0_t)$ where N^0_j is the initial prey density for experiment j.

Here we conduct Bayesian inference for parameter estimation and model selection. The likelihood of observing $n_{1:t}$ for model m with a set of parameters θ_m is denoted by $f(n_{1:t}|M = m, \theta_m, N^0_{1:t})$. Below we replace $M = m$ with just m for notational convenience. We have that $\theta_m = (a, T_h, \lambda)$ for a beta-binomial Type II or III functional response models, where a is the attack rate, T_h is the handling time and λ is a nuisance parameter required in the beta-binomial distribution to handle excess variability in the data not captured by the binomial distribution (see below for more details). Since the experiments are independent, we can write the likelihood function as:

$$f(n_{1:t}|m, \theta_m, N^0_{1:t}) = \prod_{j=1}^{t} f(n_j|m, \theta_m, N^0_j),$$

where $f(n_j|m, \theta_m, N^0_j)$ is the likelihood for the single observation n_j collected using experimental design N^0_j. For notational simplicity drop the index j for the moment. As we assume the observation n follows a beta-binomial distribution, we can write the likelihood for n as

$$f(n|m, \theta_m, N^0) = \binom{N^0}{n} \frac{B(n + \alpha, N^0 - n + \beta)}{B(\alpha, \beta)},$$

where $B(\cdot,\cdot)$ is the beta function and α and β are parameters of the beta-binomial distribution, and we now describe how they relate to the parameters θ_m of a functional response model. We define a proportion parameter p_τ and an overdispersion parameter λ such that

$$p_\tau = \frac{\alpha}{\alpha+\beta} = \frac{N^0 - N_\tau}{N^0} \text{ and}$$
$$\lambda = \frac{1}{\alpha+\beta}.$$

From the main paper, N_τ is the prey density at time τ, and depends on some of the parameters in θ_m (specifically the attack rate a and handling time T_h).

For the Bayesian approach we need to specify a prior distribution for θ_m for each model m. We denote the prior distribution for the parameter of model m as $\pi_0(\theta_m|m)$. In the main paper, we assume the components of θ_m are independent and have a log-normal distribution: $\log a \sim N(-1.4, 1.35^2)$, $\log T_h \sim N(-1.4, 1.35^2)$ and $\log\lambda \sim N(-1.4, 1.35^2)$ (the same prior as in Moffat et al. (2020)). Both models under consideration in the main paper have the same parameter, but the posterior distributions will be different between models since the underlying model formulations are different. Then we are interested in the posterior distribution of θ_m for model m after i experiments, which is given by

$$\pi_t(\theta_m|m, n_{1:t}, N^0_{1:t}) = \frac{f(n_{1:t}|m, \theta_m, N^0_{1:t})\ \pi_0(\theta_m|m)}{Z_{m,t}},$$

where $Z_{m,t}$ is the normalisation constant of the posterior (often referred to as the evidence) for model m and is given by the prior predictive probability of the observed data:

$$Z_{m,t} = f(n_{1:t}|m, N^0_{1:t}) = \int_{\theta_m} f(n_{1:t}|m, \theta_m, n_{1:t})\ \pi_0(\theta_m|m)\ \mathrm{d}\theta_m.$$

We are also interested in the posterior model probability of each model m, which assuming each model is equally likely *a priori* (as we do here), can be calculated by

$$\pi_t(m|n_{1:t}, N^0_{1:t}) = \frac{Z_{m,t}}{\sum_{k=1}^K Z_{k,t}}.$$

We cannot obtain the posterior parameter distributions and model probabilities analytically here, but we can approximate them using sequential Monte Carlo (SMC), which we describe later in Section 1.4.

1.2 Sequential Optimal Design

Sequential experimental design uses previously collected data in conjunction with a utility function to improve future data collection. We collect data points one-at-a-time and make an informed decision on the next design point. This myopic approach to experimental design has many advantages over static designs. Sequential experimental designs are generally more efficient in the presence of parameter and model uncertainty (see, for example, Dror and Steinberg, 2008) and involve lower-dimensional design optimisation problems at each iteration.

Optimal experimental design involves selecting design points such that the experimental goals are achieved in the minimum possible number of experimental runs. The experimental goals can be captured by different utility functions. In the main paper we are

interested in learning the most preferred model, but at the same time learning its parameters, as quickly as possible. This dual-purpose experimental goal depends on individual parameter estimation and model discrimination utility functions, so we explain those too in Section 1.3. Assume that we have performed t independent experiments, and thus we have available $n_{1:t}$ and $N^0_{1:t}$. Define N^0_{t+1} to be the optimal number of prey to use in the next experiment. We obtain the optimal design point by maximising the utility over the design space $\boldsymbol{N_0} \in \{1, 2, \ldots, N^0_{max}\}$ where N^0_{max} is the maximum number of initial prey that could be practically implemented in a single experiment:

$$N^0_{t+1} = \arg \max_{N^0 \in \boldsymbol{N_0}} U(N^0 | n_{1:t}, N^0_{1:t}).$$

The utility of the design N^0, $U(N^0|n_{1:t}, N^0_{1:t})$, is determined by taking the expectation of the user-specified utility function, $U(N^0, z, m|n_{1:t}, N^0_{1:t})$ (see Section 1.3), over the response and model space:

$$U(N^0|n_{1:t}, N^0_{1:t}) = \sum_{m=1}^{K} \pi_t(m|n_{1:t}, N^0_{1:t}) \sum_{z \in S} f(z|m, n_{1:t}, N^0_{1:t}, N^0) \; U(N^0, z, m|n_{1:t}, N^0_{1:t}), \tag{1}$$

where z is the number of consumed prey that might be observed using the experimental design N^0 and $S = \{0, 1, 2, \ldots, N^0_{max}\}$ is the sample space for z. The quantity $f(z|m, n_{1:t}, N^0_{1:t}, N^0)$ is the posterior predictive probability of z

$$f(z|m, n_{1:t}, N^0_{1:t}, N^0) = \int_{\theta_m} f(z|m, \theta_m, N^0) \; \pi_t(\theta_m|m, n_{1:t}, N^0_{1:t}) \; \mathrm{d}\theta_m,$$

and $\pi_t(m|n_{1:t}, N^0_{1:t})$ is the posterior probability of model m after having observed $(n_{1:t}, N^0_{1:t})$. After the optimal design N^0_{t+1} is determined, an experiment is conducted using N^0_{t+1} to reveal the observation n_{t+1}. The posterior distribution for the parameter of each model is updated in light of the new information, and the process is repeated to determine the next optimal number of initial prey to use. More details on the utility function we use in the main paper is provided below.

1.3 Utility Functions

An important component of all the utilities discussed here is the Kullback-Leibler divergence (KLD) (Kullback and Leibler, 1951). The KLD is an information-based measure of disparity between two distributions. In our case, the KLD represents the information gain on the true data generating process.

For parameter estimation of model m, the KLD between the current and updated posterior distributions of θ_m is a useful utility. For model m, the KLD between the current posterior, $\pi_t(\theta_m|m, n_{1:t}, N^0_{1:t})$, and the posterior based on the observation z and the proposed design point N^0, $\pi_{t+1}(\theta_m|m, n_{1:t}, z, N^0_{1:t}, N^0)$, is given by

$$U(N^0, z, m|n_{1:t}, N^0_{1:t}) = \int_{\theta_m} \pi_{t+1}(\theta_m|n_{1:t}, z, N^0_{1:t}, N^0) \; \log\left(\frac{\pi_{t+1}(\theta_m|n_{1:t}, z, N^0_{1:t}, N^0)}{\pi_t(\theta_m|n_{1:t}, N^0_{1:t})}\right) \mathrm{d}\theta_m, \tag{2}$$

where the dependency of the current and updated posterior on m is omitted for brevity. Equation (2) can be re-written as

$$U(N^0, z, m|n_{1:t}, N^0_{1:t}) = \int_{\theta_m} \pi_{i+1}(\theta_m|n_{1:t}, z, N^0_{1:t}, N^0) \log f(z|\theta_m, d)\ \mathrm{d}\theta_m - \log\left(\frac{Z_{m,t}(N^0, z)}{Z_{m,t}}\right), \tag{3}$$

where again the dependency of the current and updated posterior as well as the likelihood of z on m is omitted. The value $Z_{m,t}(N^0, z)$ represents the evidence at experiment number $t+1$ for model m if we pretended to observe the response z at the next design point N^0.

Alternatively, a model discrimination utility may be of interest. In this case we consider the KLD between the current and updated posterior model probability for a model m. This utility was initially suggested by Box and Hill (1967) and has been recently implemented by Drovandi et al. (2014). By ignoring terms that do not depend on the potential design N^0 for observation $t+1$, Drovandi et al. (2014) show that the utility function can be written as

$$U(N^0, z, m|n_{1:t}, N^0_{1:t}) = \log \pi(m|n_{1:t}, z, N^0_{1:t}, N^0). \tag{4}$$

The utility for design point N^0, $U(N^0|n_{1:t}, N^0_{1:t})$, is given by substituting (4) into (1).

Similar to the design problems discussed by Dette and Franke (2001), Zen and Tsai (2004) and Senarathne et al. (2020), we are interested in a dual-purpose experimental goal which combines parameter estimation and model discrimination using the total entropy criterion (Borth, 1975). Denote $U_{\mathrm{PE}}(N^0, z, m|n_{1:t}, N^0_{1:t})$ to be the parameter estimation utility from (3) and $U_{\mathrm{MD}}(N^0, z, m|n_{1:t}, N^0_{1:t})$ denote the model discrimination utility from (4). The dual-purpose utility for design point N^0 is given by

$$U(N^0, z, m|n_{1:t}, N^0_{1:t}) = U_{\mathrm{PE}}(N^0, z, m|n_{1:t}, N^0_{1:t}) + U_{\mathrm{MD}}(N^0, z, m|n_{1:t}, N^0_{1:t}), \tag{5}$$

which is purely the sum of the parameter estimation and model discrimination utilities. Through the process of simplifying and removing terms which do not depend on the design point N^0, we arrive at a dual-purpose utility:

$$\begin{aligned} U(N^0|n_{1:t}, n_{1:t}) = \sum_{m=1}^{K} \pi_t(m|n_{1:t}, n_{1:t}) \sum_{z \epsilon S} f(z|m, n_{1:t}, N^0_{1:t}, N^0) \\ \int_{\theta_m} \pi_{t+1}(\theta_m|m, n_{1:t}, z, N^0_{1:t}, N^0) \log f(z|m, \theta_m, N^0)\ \mathrm{d}\theta_m \\ - \sum_{z \epsilon S} f(z|n_{1:t}, N^0_{1:t}, N^0) \log f(z|n_{1:t}, N^0_{1:t}, N^0). \end{aligned}$$

The posterior predictive distribution $f(z|n_{1:t}, N^0_{1:t}, N^0)$ is determined by averaging $f(z|m, n_{1:t}, N^0_{1:t}, N^0)$ over all the models.

These utility functions are analytically intractable and therefore must be estimated. Unfortunately, estimating these quantities is not a straightforward process. Below we describe a sequential Monte Carlo (SMC) algorithm that is able to sequentially approximate the posterior distributions of θ_m for each m, the posterior model probabilities for each model m and the above total entropy utility function.

1.4 Sequential Monte Carlo

SMC involves traversing a set of J weighted samples (particles) for each of our K models through a sequence of slowly evolving target distributions by iteratively conducting re-weighting, resampling and move steps. We denote the set of particles representing the target for model m at experiment number t to be $\left\{\boldsymbol{\theta}^j_{m,t}\right\}_{j=1}^J$ with the corresponding normalised weights $\left\{W^j_{m,t}\right\}_{j=1}^J$. We denote the unnormalised normalised as $w^j_{m,t}$. In our implementation of SMC, the sequence of distributions is formed through data annealing, i.e. introducing data one-at-a-time. Given a particular model, m, the sequence of targets is given by

$$\pi_t(\boldsymbol{\theta}_m|m, n_{1:t}, N^0_{1:t}) \propto f(n_{1:t}|m, \boldsymbol{\theta}_m, N^0_{1:t})\ \pi_0(\boldsymbol{\theta}_m|m) \text{ for } t = 1, ..., T.$$

After an observation is collected, we re-weight the particles to reflect the updated posterior distribution. Via importance sampling arguments we obtain the following unnormalised weights

$$w^j_{m,t+1} = W^j_{m,t} f(n_{t+1}|m, \boldsymbol{\theta}^j_{m,t}, N^0_{t+1}).$$

Following this, the weights are normalised so that they sum to one and the effective sample size (ESS) is computed. The ESS refers to the number of independent samples (of equal weight) from the target distribution that the weighted sample is worth. The ESS for model m at experiment number t can be estimated by

$$\text{ESS}_{m,t} = \frac{1}{\sum_{j=1}^J (W^j_{m,t})^2}. \tag{6}$$

As more data is collected, the particle weights tend to become more skewed, producing a drop in the ESS. Thus, after an observation is introduced into each model, we check that the ESS has not dropped too much. Once the ESS drops below some threshold E, where often $E = J/2$, it indicates a lack of sample quality from the current posterior distribution for a particular model. Using such a sample can lead to estimates of quantities of interest with high variance. Therefore, to tackle this problem a resampling algorithm is used, where particles values are resampled with probabilities given by their normalised weights. This will reset the ESS back to J, but several particle values will be duplicated. Therefore, after conducting this resampling step, a move step is required to diversity the particle population.

We move each particle according to a Markov chain Monte Carlo (MCMC) kernel with invariant distribution $\pi_t(\boldsymbol{\theta}_{m,t}|m, n_{1:t}, N^0_{1:t})$. A disadvantage of using an MCMC kernel is that a move proposal might be rejected. Therefore, one iteration of MCMC on all particles may not be enough to sufficiently diversify the particle set. An appropriate number of times to conduct the move step is proposed by Drovandi and Pettitt (2011) and is given by

$$R_m \geq \frac{\log c}{\log(1-p)}. \tag{7}$$

The value $1 - c$ is a pre-specified probability that the particle will move and p is the probability of acceptance at the MCMC move step (here we set $c = 0.01$). This acceptance probability, p, is estimated by conducting one MCMC move step for each particle in the set and determining the overall proportion of particles which move.

A useful property of this algorithm is that for each model m, we can approximate the log evidence, $\log Z_{m,t}$, using the particle weights. Del Moral et al. (2006) show that we can approximate the ratio of normalising constants, $Z_{m,t+1}/Z_{m,t}$, and hence the posterior predictive distribution, $f(n_{t+1}|m, n_{1:t}, N^0_{1:t+1})$, for each model at the current experimental number t using

$$Z_{m,t+1}/Z_{m,t} = f(n_{t+1}|m, n_{1:t}, N^0_{1:t+1}) \approx \sum_{j=1}^{J} W^j_{m,t} f(n_{t+1}|m, \boldsymbol{\theta}^j_{m,t}, N^0_{t+1}).$$

Since we know that $\log Z_{t+1} = \sum_{v=0}^{t} \log(Z_{t+1-v}/Z_{t-v})$ and $Z_0 = 1$, we can easily approximate $\log Z_{t+1}$ in our SMC algorithm. This can be achieved via the following recursion: $\log Z_{m,t+1} = \log Z_{m,t} + \log(Z_{m,t+1}/Z_{m,t})$.

1.5 Estimation of Utility Functions

Now we demonstrate how we can use the output of SMC to approximate the total entropy utility function. Define $w^j_{m,t}(N^0, z)$ and $W^j_{m,t}(N^0, z)$ to be the updated unnormalised and normalised weights of the jth particle after observing the number of prey attacked z at design N^0, respectively. We estimate the ratio of two normalising constants and hence the posterior predictive distribution by

$$\frac{Z_{m,t}(N^0, z)}{Z_{m,t}} = f(z|m, n_{1:t}, N^0_{1:t}, N^0) \approx \sum_{j=1}^{J} W^j_{m,t} f(z|\boldsymbol{\theta}^j_{m,t}, N^0) = \sum_{j=1}^{J} w^j_{m,t}(N^0, z). \qquad (8)$$

Using the normalised weighted samples and Monte Carlo integration, we can approximate the integral within the parameter estimation utility in (3):

$$\int_{\boldsymbol{\theta}_m} \pi_{t+1}(\boldsymbol{\theta}_m|m, n_{1:t}, z, N^0_{1:t}, N^0)\ \log f(z|m, \boldsymbol{\theta}_m, N^0)\ \mathrm{d}\boldsymbol{\theta}_m \approx \sum_{j=1}^{J} W^j_{m,t}(N^0, z) \log f(z|m, \boldsymbol{\theta}^j_{m,t}, N^0).$$

We estimate the posterior predictive distribution, $f(z|n_{1:t}, N^0_{1:t}, N^0)$, by

$$f(z|n_{1:t}, N^0_{1:t}, N^0) \approx \sum_{m=1}^{K} \hat{\pi}_t(m|n_{1:t}, N^0_{1:t}) \sum_{j=1}^{J} w^j_{m,t}(N^0, z).$$

The posterior model probabilities at experiment number t, $\pi_t(m|n_{1:t}, N^0_{1:t})$, can be estimated by normalising the estimated evidences. Using these approximations together with (1), we can approximate the parameter estimation and model discrimination utilities, and hence the total entropy. The SMC algorithm for optimal sequential experimental design is presented in algorithm 1.

2 Simulation Study

In the simulation studies in Moffat et al. (2020), four candidate models are considered; the Type II and III functional response models with either a binomial or beta-binomial response distribution. Here we conduct some further simulation studies to assess if there is any consequence of considering only two candidate models, i.e. removing the two binomial

Algorithm 1 SMC algorithm for sequential experimental design

INPUT: Total number of experiments to run, T, number of samples for each model, J, an appropriate ESS threshold, E, the model prior distributions, $\pi_0(\boldsymbol{\theta}_m|m)$, and the likelihood function, $f(n_t|m, \boldsymbol{\theta}_m, N_t^0)$ for each model m.

OUTPUT: The selected initial number of prey to use for the experiments, $N_{1:T}^0$ and the corresponding number of prey attacked, $n_{1:T}$.

Draw samples from model priors, $\boldsymbol{\theta}_{m,0}^j \sim \pi_0(\boldsymbol{\theta}_m|m)$, for $m = 1, ..., K$ and for $j = 1, ..., J$.
Initialise weights, $W_{m,0}^j = 1/J$, for $m = 1, ..., K$ and for $j = 1, ..., J$.
Initialise log evidences, $\log \hat{Z}_{m,0} = 0$, for $m = 1, ..., K$.
for $t = 0$ to $T - 1$ **do**
  Select design point N_{t+1}^0 to maximise some given utility $U(N^0|n_{1:t}, N_{1:t}^0)$.
  Collect data n_{t+1} by performing an experiment based on N_{t+1}^0.
  for $m = 1$ to K **do**
    Compute the updated unnormalised weights, $w_{m,t+1}^j = W_{m,t}^j f(n_{t+1}|m, \boldsymbol{\theta}_{m,t}^j, N_{t+1}^0)$, for $j = 1, ..., J$.
    Update the log evidence, $\log \hat{Z}_{m,t+1} = \log \hat{Z}_{m,t} + \log \sum_{j=1}^{J} w_{m,t+1}^j$.
    Normalise the weights, $W_{m,t+1}^j = w_{m,t+1}^j / \sum_{q=1}^{J} w_{m,t+1}^q$, for $j = 1, ..., J$.
    Compute the effective sample size, $\text{ESS}_{m,t+1} = 1/\sum_{j=1}^{J}(W_{m,t+1}^j)^2$.
    if $\text{ESS}_{m,t+1} < E$ **then**
      Resample particle set to obtain $\left\{\boldsymbol{\theta}_{m,t+1}^j\right\}_{j=1}^{J}$.
      Set $W_{m,t+1}^j = 1/J$ for $j = 1, ..., J$.
      Determine the parameters of the MCMC proposal $q_{m,t+1}(\cdot|\cdot)$ using the current particles, $\left\{\boldsymbol{\theta}_{m,t+1}^j\right\}_{j=1}^{J}$.
      for $j = 1$ to J **do**
        Conduct a one iteration move step by moving the particle $\boldsymbol{\theta}_{m,t+1}^j$ with an MCMC kernel of invariant distribution $\pi_{t+1}(\boldsymbol{\theta}_{m,t+1}|m, n_{1:t+1}, N_{1:t+1}^0)$.
      end for
      Calculate acceptance probability, p, and hence R_m.
      for $j = 1$ to J **do**
        Move particle $\boldsymbol{\theta}_{m,t+1}^j$ with an MCMC kernel of invariant distribution $\pi_{t+1}(\boldsymbol{\theta}_{m,t+1}|m, n_{1:t+1}, N_{1:t+1}^0)$ iterated $R_m - 1$ times.
      end for
    else
      Set $\boldsymbol{\theta}_{m,t+1}^j = \boldsymbol{\theta}_{m,t}^j$ for $j = 1, ..., J$.
    end if
  end for
end for

response models from the candidate set. By considering only one response model it might be possible to more quickly discriminate between the Type II and III functional response models.

Firstly we delve into a scenario where the sequential design algorithm struggles to discriminate between the beta-binomial and binomial models. Consider the following simulation study. Set the Type II beta-binomial model as the true model and include all four previously mentioned models in the candidate set. Create a list of 10 parameter configurations by fixing $\lambda = 0.02$ and sampling a and T_h from a posterior distribution based on the data from Papanikolaou et al. (2016). Set the minimum and maximum designs to be 1 and 130, respectively. Let the total exposure time of prey and predator be 8 hours. Using the same prior distribution defined in section 1.1, apply the sequential design algorithm with each parameter configuration for 30 observations. Repeat the algorithm 30 times for each configuration. To find optimal designs, use the total entropy utility function where λ is treated as a nuisance parameter.

Figure 1 compares the average (averaged over all of our simulations) posterior model probability of each candidate model over the 30 sequential design iterations. For the first couple of observations, the binomial models are preferred due to the complexity of the beta-binomial models. However, as more data is collected, the overdispersion in the data becomes more apparent and thus the posterior model probability for the beta-binomial models begin to increase.

It is evident from Figure 1 that when the true lambda parameter is small, a significant amount of experimental effort is spent on trying to discriminate between the binomial and beta-binomial variations. Even at the conclusion of the data collection process (30 observations), discriminating between the binomial and beta-binomial models is difficult. This is because the binomial model is a special case of the beta-binomial model, and thus the beta-binomial model is able to mimic what the binomial model can do. Eventually, either the beta-binomial model will be discarded as it will be penalised for its complexity, or the binomial model will be discarded as it is unable to explain the overdispersion in the data. In either case, we generally find that a significant amount of experimentation is required to reach either conclusion. This is quite wasteful as we are more interested in discriminating between the Type II and III models, rather than which response distribution is correct. Although this is only one example, it is clear that given certain true parameters, discriminating between the beta-binomial and binomial variations can be troublesome, particularly on a budget.

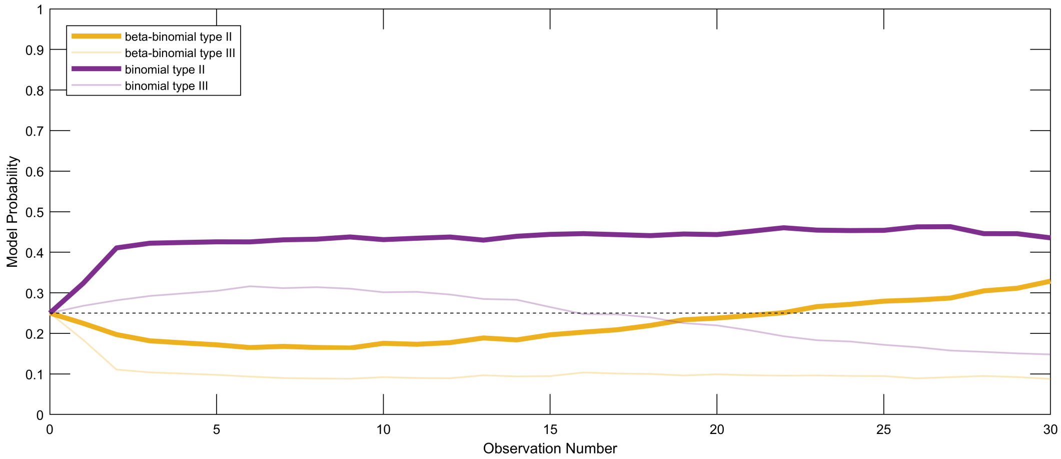

Figure 1: The average posterior model probability for each of the candidate models. The true model is Model 1 (beta-binomial Type II). Each of the true parameter configurations considered have a λ value of 0.02.

Therefore we propose to consider models with only a beta-binomial response distribution, as this also includes the binomial response models as a special case. We conduct another simulation study to assess the negative impact on parameter estimation of a and T_h when the binomial response models are true but we do not include them in the candidate list. We run a total of 600 simulated sequential designs. This comprises of 30 repetitions of 10 different parameter configurations for each of the binomial models. Any setting that is not mentioned here is set to be the same as the preceding simulation study.

Figure 2 displays the proportion of simulations for which the parameter values from our true binomial model are within a 95% credible interval of the posterior of our beta-binomial model. Since the proportions are relatively high, we lose very little in terms of parameter estimation accuracy by assuming the beta-binomial response distribution. In light of this simulation study, for the real experiments, we do not consider the binomial response models in the candidate set.

```
################################################################
8 HOUR EXPERIMENTS 30 observations, prey densities up to 130
################################################################

True Model: Type II Binomial
Proportion of Binomial True Values in Beta-Binomial 95 percent credible interval:
a                     Th
0.956667              0.976667

-------------------------------------------------------------------
True Model: Type III Binomial
Proportion of Binomial True Values in Beta-Binomial 95 percent credible interval:
a                     Th
0.903333              0.993333
```

Figure 2: Results from simulation study.

References

Borth, D. (1975). A total entropy criterion for the dual problem of model discrimination and parameter estimation. *Journal of the Royal Statistical Society. Series B (Methodological)*, 37(1):77–87.

Box, G. and Hill, W. (1967). Discrimination among mechanistic models. *Technometrics*, 9(1):57–71.

Del Moral, P., Doucet, A., and Jasra, A. (2006). Sequential Monte Carlo samplers. *Journal of the Royal Statistical Society: Series B (Statistical Methodology)*, 68(3):411–436.

Dette, H. and Franke, T. (2001). Robust designs for polynomial regression by maximizing a minimum of D-and D1-efficiencies. *The Annals of Statistics*, 29(4):1024–1049.

Dror, H. and Steinberg, D. (2008). Sequential experimental designs for generalized linear models. *Journal of the American Statistical Association*, 103(481):288–298.

Drovandi, C., McGree, J., and Pettitt, A. (2014). A sequential Monte Carlo algorithm to incorporate model uncertainty in Bayesian sequential design. *Journal of Computational and Graphical Statistics*, 23(1):3–24.

Drovandi, C. and Pettitt, A. (2011). Estimation of parameters for macroparasite population evolution using approximate Bayesian computation. *Biometrics*, 67(1):225–233.

Kullback, S. and Leibler, R. (1951). On information and sufficiency. *The Annals of Mathematical Statistics*, 22(1):79–86.

Moffat, H., Hainy, M., Papanikolaou, N., and Drovandi, C. (2020). Sequential experimental design for predator–prey functional response experiments. *Journal of The Royal Society Interface*, 17(166):20200156.

Papanikolaou, N., Demiris, N., Milonas, P., Preston, S., and Kypraios, T. (2016). Does mutual interference affect the feeding rate of aphidophagous coccinellids? A modeling perspective. *PLOS ONE*, 11(1):e0146168.

Senarathne, S., Drovandi, C., and McGree, J. (2020). Bayesian sequential design for Copula models. *TEST*, 29:454–478.

Zen, M.-M. and Tsai, M.-H. (2004). Criterion-robust optimal designs for model discrimination and parameter estimation in fourier regression models. *Journal of Statistical Planning and Inference*, 124(2):475–487.
