# Supplementary material for: Adaptive experimental design produces superior and more efficient estimates of predator functional response
Source: PLoS One. 2023 Jul 20;18(7):e0288445. doi: 10.1371/journal.pone.0288445 (PMC10358903; doi:10.1371/journal.pone.0288445)
Supplement: Attachment — Submitted filename: Response_To_Referees.pdf [file pone.0288445.s002.pdf]

Response to Editor

Personally, I read your manuscript with pleasure. I think that your proposed approach has a potential to become a standard since it is more cost-effective than standard designs. It is useful and general as it could accommodate more complex functional responses, for example with predator interference.

Response: We thank the editor for their time for handling this manuscript, and for their encouraging feedback.

As you will see, both Reviewers are positive about your contribution but they also raise a number of comments that could be handled without difficulty. The main issue is raised by Reviewer 2 and concerns the logic of sequential designs. This is a valid remark that, to my opinion, questions more the general approach of sequential designs and not specifically your contribution. However, it is important and useful to explain how Bayesian inference differs from the frequentist approach in dealing with parameter estimation, and potentially how it can account for random factors.

Response: Please see below for point-by-point response to each of the referee's comments. In addressing the comments, we hope to have allayed any fears on the statistical soundness of the adaptive design approach. To clarify how the Bayesian approach works, we have added the following to the paper: " **Our methods are Bayesian, and we thus treat the model indicator as well as the parameters of each of the models as random variables. Before collecting any data, we allocate these quantities prior distributions."** After each observation is collected, we produce estimates of the posterior model probabilities for each model and the posterior distributions for the parameters of each candidate model." See lines 130-134 for the changes.

Response to Referee 1

This paper describes a procedure for optimal design of functional response experiments, designed to achieve best estimates with the fewest number of experiments / replicates, using Bayesian inference and Monte Carlo simulations. The procedures are derived from previous papers (particularly: Moffat H, et al. 2020 Sequential experimental design for predator–prey functional response experiments. J. R. Soc. Interface 17: 20200156. http://dx.doi.org/10.1098/rsif.2020.0156, and related empirical papers). The "adaptive" aspect of the design involves using preliminary experiments sequentially to improve model selection or estimation by not only adding more replicates but optimally choosing the levels of the independent variable (number of prey offered) that most efficiently achieve the goals of the study. They paper advances what is shown in that previous paper by illustrating how the procedure works with real experimental data, as opposed to simulation that were used in the previous paper. The current paper shows how the goals of experiments may be met with fewer experiments, and how estimates may be improved using this adaptive experimental designs.

My background is that of an ecologist with some background in applied, non-Bayesian statistics, but limited background in mathematical / statistical theory. Hence, I will have relatively little to say about the technical aspects of the optimization methods used and the mathematical basis of this approach. However, the overall goal of more efficient experiments is an important one for empiricists, and I believe this paper makes a sound case for the utility of the adaptive approach. Some of my suggestions are directed at making the presentation clearer, particularly for those who may not have the strong mathematical background evident in the paper and supplement, but who nevertheless are interested in using experiments on functional responses to answer biological questions. My hope would be that those suggestion would make it more likely that this adaptive approach becomes commonly used.

Response: We thank the referee for their time, positive feedback on the manuscript, thoughtful and constructive comments for improving the paper. We address the specific comments below.

Major comments.

Line 128-129. Regarding p(tau). This statement would mean that p(tau) = proportion of prey eaten for experiment of duration tau, p(tau) =n/N0. Correct?

Response: We agree with the referee that the way we have written this is a bit confusing. We have now provided more detail on how we convert the deterministic model into a probabilistic model that should hopefully be more clear. See lines 106-111.

Lines 138-141. When I first read this, I interpreted it to mean that the independence assumption means that the experiments are conducted individually, in sequence, at different times, using a single N0 at each time. Based on later statements (Line 190), I believe I read too much into this description. Sequential, at different times, but flexible about the number of different N0 values. Think you could make your meaning clearer here.

Response: The notation in this part is more generic, and could apply if the data were collected sequentially or in parallel. We have added the following to make this more clear: "To precisely

estimate the parameters and discriminate between the models, multiple independent experiments are needed**, collected either sequentially or in parallel, with a potentially different prey density used for each individual experiment.**" See lines 119-121.

Line 213, Figure 4. This figure needs a better, more explicit caption. Is there any connection between these posterior distributions and the bivariate distributions shown in Fig. 6? See further comment on Figure 6.

Response: We have now added more information to the caption, making it explicit that these are posterior distributions based on the pilot experiment data only. Figure 6 are posterior distributions based on all the collected data for a particular design choice (i.e. either baseline or sequential). See Figure 6 caption.

Lines 222-223. In addition to deciding on the model most appropriate for a predator's feeding (type II or III), and estimating that model's parameters, another goal of many functional response studies is to compare statistically 2 or more functional responses to test the hypotheses that one or more FR parameters differ among the groups (e.g., males vs. females). My guess is that if your approach produces better estimates, then it should produce better, more statistically powerful, comparisons. I think you should address this issue if you can. It seems like this comparison of males vs. females in the data you use would allow you to address that issue.

Response: It turns out for this study that the parameter values between males and females are quite different, so that even the baseline design approach is sufficient for obviously noticing a difference in parameter values (little overlap in 90% credible regions between males and females). Note also that our adaptive approach for this study did not explicitly aim to test if there is a difference between males and females; we essentially treated males and females as separate studies. Thus we do not necessarily expect our adaptive approach to produce more powerful comparisons in this respect in this particular case. But it would be possible to incorporate this within our design approach if it was of interest. We have added the following to the revised manuscript to summarise this (see lines 230-241):

"In terms of comparing whether there is a difference in parameter values between males and females, it can be seen from Figure 6 that there is little overlap in the 90% credible regions between males and females, regardless of the experimental design used (i.e. baseline or adaptive). Thus, for this predator-prey system, there is strong evidence of a difference in parameter values between males and females. It is important to note that in this study we did not explicitly aim to design for testing if there was a difference in parameter values between males and females; we essentially treated males and females as separate studies. If there was interest in designing to distinguish between the parameters of males and females, then additional models should be incorporated into the candidate list that assume shared parameter values between males and females (as well as the models that allow parameter values to be different between males and females)."

If you prefer to cast this in a model selection context, rather than hypothesis testing, then address whether your approach improves your ability to decide e.g., whether a full model with same parameters (a, Th) for males and females is preferable to models postulating that one or both parameters (a, Th) have different values for males and females, as indicated by model likelihood or information criteria. My main point is that COMPARISONS are another issue your paper should address.

Response: We agree with this point and it is one we would like to address in future experiments, because another approach is required (please see response to above comment).

Line 239, Figure 6. I think Figure 6 needs greater explanation, particularly exactly what the concentric isoclines represent - Empirical distributions? Bootstrapped values? confidence intervals? A more detailed explanation will make your point more clearly even to people who don't think Bayesian

Response: We have now provided more information in the caption on how to interpret the contour plots (see Figure 6 caption).

Figure 6 caption. For males this appears to be centered on Th=0.2 and a=0.22 That does not seem to match well with what is shown in Figure 4 for the univariate distributions after the pilot expt. Is that because these estimates improved (and shifted) compared to the pilot? The same mismatch is evident in the female data. I think your point would be clearer if you showed Figure 4 (after pilot) and Figure 6 (Final) distributions the same way, and likely side by side. I like the bivariate form. This would make it clearer what the reader is seeing and showing the improvement of the estimates that occurs via the adaptive design. You might show that on the same graph, but that would need to be separated from the comparison of adaptive vs. static.

Response: As suggested, in Figure 4 we have replaced the marginal posterior estimates with the bivariate estimates. We have also overlaid the adaptive design contours for reference. There is a shift in the mode between the pilot and adaptive data as the data provide more information about the true underlying parameter values, however the posterior is still well within the support of the prior, so there isn't any prior-data conflict to be concerned about.

Discussion and Supplementary material. I think that in some cases, running the experiments sequentially may create a confounding problem, if the predators, prey, conditions, or experimenters vary over time. That would mean that different prey densities (implemented in experiments at different times) would be confounded with different time effects. Whether this is a real problem, and how serious it is would likely depend on the details of the experiment (the organisms, the set up, and how far apart in time the experiments are run). Have you considered this issue and is there a way to detect it while implementing the optimal design? The different iterations of the experiments performed at different times seem to me to be equivalent to temporal blocks. Could the optimal design incorporate something like temporal blocks if the time effect does prove to be a non-trivial issue? I suspect that for the system you describe here, this is not much of an issue, with duration of only 8 h, and fairly short-lived and likely numerous prey and predators. But in other systems, with longer experimental times, or prey / predators that are more difficult to acquire in large numbers (which might actually be the kinds of organisms where this more efficient design is most valuable) the temporal effect might be more important. Some discussion of this issue would be useful, in the main paper.

Response: We agree with the referee that more discussion on how the adaptive approach can extend to more complicated models is needed, and also discussion about potential time effects. We have added the following paragraph (see lines 289-300):

"This study considered relatively simple stochastic models of predator-prey systems. However, in principle, more complex models can be accommodated in our framework, such as those modelling predator interference (Skalski and Gilliam 2001). For experiments conducted in different spatial locations, a fixed or random spatial effect can be added to the model. Our experiments were conducted over a short time period. However, for systems with longer experimental times, various conditions and the manner in which the predator and prey interact may change. In which

case, it might be necessary to allow parameters to be time-varying, permitting small changes in either continuous or discrete time. We do acknowledge that the optimal approach to experimental design can become more computationally intensive for more complicated models (see Ryan et al 2016 for a detailed discussion of the issues). Importantly, the adaptive design approach requires fewer runs to achieve the statistical goals, which can help to mitigate potential temporal effects. "

Supplementary material. My mathematical expertise does not allow me to critique the programming or modeling approach used here. The code mentioned in the preliminary information in the manuscript itself will be very useful in enabling ecologists with lesser mathematical / coding experience to implement these methods. The Github code sets (for R and Matlab) from the previous paper by Moffat et al. are a good model for the kind of detail that would help experimentalists. The more you can provide, the more often and widely your approach will be implemented.

Response: We have placed the data and Matlab code on a GitHub repository and linked to it in the paper (see lines 170-172).

Minor comments.

Line 68. Spelling. The name is "Stouffer"

Response: We apologise for getting this wrong, we have fixed it now.

Line 80. 'among' would be a better word. A restructured sentence might be clearer: "...that it would be challenging to discriminate among these three functional forms..."

Response: We have changed our explanation for figure 1 as per another referee comment (see lines 79-84).

Line 119. Th . check your symbols.

Response: We thank the referee for spotting this, it has now been fixed throughout the paper.

Line 198 (and multiple other places, e.g., line 285). "data" is a plural, so 'are' should replace 'is'.

Response: Changed as suggested throughout the paper.

Line 209. Missing figure number. Figure 4 is what you mean here, I believe. This also comes up elsewhere (line 228, 239), perhaps as a problem translating to PDF?

Response: We think this might be a result of some Figures being dynamically linked, and then the convert to pdf. So we have used all manual references to figures now.

Line 240. 'have' not 'has'

Response: Fixed.

Response to Referee 2

This is a very well written and clear manuscript. The authors do an excellent job of explaining and complex and potentially powerful approach. I find the idea of adaptive experimental designs intriguing and potentially powerful. I was particularly compelled by the idea that this could reduce the number of animals needed for experiments. However, there are a few places where I think the manuscript could be made clearer and a few big statistical questions that still need to be addressed.

Response: We thank the referee for their time, positive feedback on the manuscript, and for the thoughtful and constructive comments for improving the paper. We address the specific comments below.

Line 74-76: I do not think this description of a typical experimental design is accurate. I would argue that most studies use a static experimental design where a set of predators are provided one of several densities and all density treatments are conducted simultaneously. The iterative approach described is not the typical method. More justification of this is needed or clarification of when such designs are used relative to more typical experimental designs.

Response: We agree that what we described is not general enough to encompass what is traditionally done. We have changed it to the following "The consumed number of prey out of the total that the predator has access during that given time period is recorded. This process is then repeated independently of previous experiments, either in parallel or sequentially as resources permit, for a total of *T* experiments. Each individual experiment is assigned a given initial prey density from a set of initial prey densities to be tested." (see lines 34-39)

Line 79-81: I feel that this example is a bit of a straw man. We do not typically use functional responses to distinguish models at a single high or low density as depicted in Figure 1. Instead, inferences are more typically based on the entire Functional response curve, or the curves are used to make projections. In the latter case predictions at density B would be indistinguishable and the implications minimal for population dynamics.

Response: We do not mean to imply that data would only be sampled at a single prey density, and we see how the format of Figure 1 might suggest this. We have therefore revised the figure and associated text to highlight the issue better (see lines 42-47):

"In Figure 1, for example, experiments undertaken at the prey level marked (A) can more easily differentiate the Type III functional response from types I and II. By contrast, experiments undertaken at the prey level marked (B) can more easily differentiate the Type I functional response. As experimental data begins to accumulate, it is possible that one of these two levels of prey density offers more information; a non-optimal experimental design cannot adapt to this fact."

Material and Methods:

The description of the functional response experiments on line 102 – 110 was inadequate. It was not clear that there were eggs on the tomato leaflet, what densities were used, and how many replicates. This information is not presented until > 70 lines later. I think the design and contrasts

would be more clear if the presentation was reorganized to highlight the baseline and pilot design first and then describe the adaptive methodology. Or describe the method first and then the full design used to test it.

Response: As suggested by the referee, we have moved the description of the baseline design and data earlier to where the experiment is described. We have also provided more detail on the experiment itself in the "Functional response data" section, but also throughout other parts of the paper. (see Section "Functional response data")

Discussion:

I feel like this paper is incomplete without formal analysis of the approaches suggested on lines 284 – 288. These intermediate designs are most relevant for most researchers and systems. This might also allay some of the concerns outlined in the next section.

Response: As already mentioned in the manuscript, a comprehensive comparison of the optimal static and adaptive design approaches has already been conducted via simulation studies in our previous work (Moffat et al 2020). Intuitively, the adaptive approach will on average perform better as it makes more informed decisions as data are collected sequentially (as we mention in the paper). Whereas the optimal static design only uses information from the original prior distribution.

General Comment:

My biggest concern with the presentation is the lack of statistical exploration and explanation. In a typical static experimental design, all density treatments are carried out simultaneously and are presumable randomly assigned to predators. The entire set of density treatment may then be replicated in multiple replicate blocks. The effects of time, spatial location, or other variable that might have affected the specific set of predators and prey used in a replicate can be estimated and accounted for as a random effect. How does this piecemeal approach estimate and propagate these types of errors, and the nonrandom assignment of treatments?

Response: Adaptive, or sequential, experimental design is a principled approach that has been well studied in the statistical literature. In terms of practical application, it is commonly used in clinical trials. If there are other effects that impact on the response then they should be included in the statistical model that is being designed for. For example, if there are spatial effects, then they should be included in the model. The adaptive design approach is agnostic to the statistical models that have been proposed for the data that will be collected. The assignment of treatments are technically random, since they depend on the sequentially collected data, which is itself random. We have added the following to the manuscript to allay any fears about the adaptive experimental design approach not being statistically sound: **"Bayesian adaptive experimental design methods have been comprehensively studied in the statistical literature (see Ryan et al. (2016) for a review), and are commonly used in clinical trials (e.g. Giovagnoli (2021))."** (see lines 162-164)

We agree with the referee that more discussion on how the adaptive approach can extend to more complicated models is needed. We have added the following paragraph to the Discussion (see lines 289-300):

"This study considered relatively simple stochastic models of predator-prey systems. However, in principle, more complex models can be accommodated in our framework, such as those modelling

predator interference (Skalski and Gilliam 2001). For experiments conducted in different spatial locations, a fixed or random spatial effect can be added to the model. Our experiments were conducted over a short time period. However, for systems with longer experimental times, various conditions and the manner in which the predator and prey interact may change. In which case, it might be necessary to allow parameters to be time-varying, permitting small changes in either continuous or discrete time. We do acknowledge that the optimal approach to experimental design can become more computationally intensive for more complicated models (see Ryan et al 2016 for a detailed discussion of the issues).”

These issues became particularly prescient for me as I examined Figure 2. This sequential adaptive process looks alarmingly like a phenomenon commonly referred to as p-hacking in traditional hypothesis testing. How is this method different from ad hoc experimental design which occurs when you did not choose a sample size a priori, but instead keep doing replications until you get desired results. I realize the proposed methods are much more sophisticated than a simple hypothesis test. However, it still seems as though there needs to be some sort of penalty that you must pay for the adaptive “adaptive“ sample size decisions prior to declaring a “significant” or best model.

Response: It was interesting to think about the difference between adaptive design and p-hacking. The major difference with p-hacking, is that in p-hacking there is a particular hypothesis in mind that the researchers are hoping to be true. In adaptive design, a potential stopping rule is when there is a lot of evidence in favour of one of the models, but we don’t specify beforehand which model we are hoping is the one revealed. Note that it is not essential to implement a stopping rule (we didn’t do so in our paper, we decided a priori how many experiments we will run), but it can be beneficial to save on resources when a certain amount of the “truth” has revealed itself. One penalty that is paid, as discussed in the manuscript, is that when data are collected sequentially, compared to in parallel, it may take longer to collect the data.
