# Supplementary material for: Adaptive experimental design produces superior and more efficient estimates of predator functional response
Source: PLoS One. 2023 Jul 20;18(7):e0288445. doi: 10.1371/journal.pone.0288445 (PMC10358903; doi:10.1371/journal.pone.0288445)
Supplement: Attachment — Submitted filename: Response_To_Referees_Revision.pdf [file pone.0288445.s003.pdf]

Response to Editor

We thank the editor for the very careful reading of the revised paper and for the suggestions as well as the identified typos. Please see below a point-by-point response to each of the comments.

1) Legend of Fig. 2: I would be clearer with the abbreviations of the figure (a figure should be understandable without reference to the main text). I suggest:

"In Step 1 some pilot experiments are conducted by varying the prey density N and measuring the number of prey eaten n. They are combined with the prior information to obtain the posterior distribution for the model parameters (Omega = m1 and m2) and model probabilities (p) in Step 2. Assuming that the experimental goals have not been met with the pilot data, the adaptive design process begins in Step 3 where the optimal prey density (N) to use for the next experiment is determined by maximising the utility function U(N). In Step 4 the experiment is conducted using the prey density found from Step 3. The data collected from Step 4 is then fed back to Step 2 to update the posterior distributions. We again check if the experimental goals have been collected or if the experimental resources have been consumed. If so, we stop the experiment and report the final Bayesian inferences, otherwise we continue to iterative the adaptive design process (Steps 3, 4 and 2 in that order)."

Please check my proposition for consistency and format if necessary. Also, in the box for Step 4, I would add a vertical arrow from N=11 pointing to n=3 to make evident that n=3 is the result of the experiment.

Response: We have changed the figure caption as suggested. We have also included an arrow in the box for Step 4 as suggested.

2) line 183: Remove the second of in "...the number of pilot of experiments."

Response: Done

3) lines 188 to 193: I would provide a reference for the "log determinant of the posterior covariance matrix", as this concept may be unknown for the majority of readers (it was for me).

Response: A reference has been added

4) line 195: Remove "produces data" in "The data produced by the baseline experimental design produces data are shown in Figs..."

Response: Done

5) lines 200-202: "The posterior model probabilities for males and females are 0.581 and 0.559 respectively, in favour of a Type II functional response."

First, I would use "model 1" and "model 2" everywhere in the Result section. Second, it is not clear to me why the model 1 is favoured (see also lines 214-215). It seems to me that the information should be given in Fig. 5, but I do not see the results for model 2 in this figure. Please clarify this issue.

Response: In the results we now only refer to Model 1 and Model 2 instead of Type II and Type III, respectively. We have added "(since there are only two models)" to demonstrate why Model 1 is preferred (since Model 1's probability is above 0.5, which means that Model 2's probability is less than 0.5).

6) line 209 and Fig. 3: please check the identity of the four panels in the text and in the figure. Moreover, the panel titles do not correspond to the legends.

Response: We now refer to the panel labels and also provide more information in the figure caption on what is shown in each panel.

7) Legend of Fig. 4 and Fig. 6: The "top and bottom rows" do not correspond to the figures. Please use instead "panel a" and "panel b".

Response: We now refer to the panels.

8) Legend of Fig. 5: Please correct the first sentence ("Shows are results for...").

Response: "Shows" changed to "Shown"

9) Legend of Fig. 6: Rather than repeating the long text of the legend of Fig. 4, I would simply use "See the legend of Fig. 4 for information on the contour lines".

Response: Done
